# Immunogenomic Landscape in Breast Cancer Reveals Immunotherapeutically Relevant Gene Signatures

**DOI:** 10.3389/fimmu.2022.805184

**Published:** 2022-01-27

**Authors:** Tao Wang, Tianye Li, Baiqing Li, Jiahui Zhao, Zhi Li, Mingyi Sun, Yan Li, Yanjiao Zhao, Shidi Zhao, Weiguang He, Xiao Guo, Rongjing Ge, Lian Wang, Dushan Ding, Saisai Liu, Simin Min, Xiaonan Zhang

**Affiliations:** ^1^ College of Life and Health Sciences, Northeastern University, Shenyang, China; ^2^ Department of Immunology, Bengbu Medical College, Bengbu, China; ^3^ Department of Pathophysiology, Bengbu Medical College, Bengbu, China; ^4^ Department of Radiology, Tian Jin Fifth’s Central Hospital, Tianjin, China; ^5^ College of Pharmacy, Beihua University, Jilin, China

**Keywords:** immune subtype, tumor microenvironment, immune escape, immunotherapy, breast cancer

## Abstract

Breast cancer is characterized by some types of heterogeneity, high aggressive behaviour, and low immunotherapeutic efficiency. Detailed immune stratification is a prerequisite for interpreting resistance to treatment and escape from immune control. Hence, the immune landscape of breast cancer needs further understanding. We systematically clustered breast cancer into six immune subtypes based on the mRNA expression patterns of immune signatures and comprehensively depicted their characteristics. The immunotherapeutic benefit score (ITBscore) was validated to be a superior predictor of the response to immunotherapy in cohorts from various datasets. Six distinct immune subtypes related to divergences in biological functions, signatures of immune or stromal cells, extent of the adaptive immune response, genomic events, and clinical prognostication were identified. These six subtypes were characterized as immunologically quiet, chemokine dominant, lymphocyte depleted, wounding dominant, innate immune dominant, and IFN-γ dominant and exhibited features of the tumor microenvironment (TME). The high ITBscore subgroup, characterized by a high proportion of M1 macrophages:M2 macrophages, an activated inflammatory response, and increased mutational burden (such as mutations in TP53, CDH1 and CENPE), indicated better immunotherapeutic benefits. A low proportion of tumor-infiltrating lymphocytes (TILs) and an inadequate response to immune treatment were associated with the low ITBscore subgroup, which was also associated with poor survival. Analyses of four cohorts treated with immune checkpoint inhibitors (ICIs) suggested that patients with a high ITBscore received significant therapeutic advantages and clinical benefits. Our work may facilitate the understanding of immune phenotypes in shaping different TME landscapes and guide precision immuno-oncology and immunotherapy strategies.

## Introduction

Human breast cancer remains a threat to women’s health worldwide and is characterized by intra-tumoral heterogeneity with biological and clinical diverseness (1, 2). Several lines of clinical evidence indicate that refined molecular taxonomy helps breast cancer prognostication and therapeutic stratification (3, 4). Some excellent studies have defined tumor subtypes using various methods, such as histopathological classification methods (based on ER, PR, and HER2), gene expression-based classification methods (PAM50) (5), and immunogenomics methods (6), which have been conducive to the identification of novel therapeutic targets. Different subtypes of breast cancer present distinct tumor microenvironment (TME) characteristics, especially adaptive immunity. Although the properties, immunoediting ability, and diversity of the T cell receptor (TCR) repertoire of the adaptive immune response have been explored (6–9), the extensive immune landscape of breast cancer has not been fully elucidated.

In the past decade, cancer immunotherapy has significantly revolutionized the management of cancer. By utilizing the patient’s immune system to identify and control tumors, immune checkpoint inhibitors (ICIs) specific for PD-1, PD-L1, and CTLA-4 have successfully enhanced the outcomes of various malignancies (10). However, recent clinical trials have demonstrated that immunotherapies showed low efficiency in the entire population of breast cancer patients, especially those with triple-negative breast cancer (TNBC) (11). Except for the fact that breast cancer exhibits similar immunologically silent properties, a low mutational burden, and fewer tumor-infiltrating lymphocytes (TILs) than some other cancers (12, 13), the lack of an immunogenomic approach for appropriate patient selection and precise prognostic biomarkers might be the mainspring for these discouraging results.

This study took the form of an integrative immunogenomic analysis to characterize the immune TME in breast cancer and explored what genomic events contribute to these results. With multi-omics data from The Cancer Genome Atlas (TCGA) database and immune gene expression signatures, we first classified breast cancer samples into six immune subtypes that exhibit distinct immune escape mechanisms and provide promising therapeutic and prognostic implications for cancer treatment. We also established a robust predictive signature to estimate the outcomes of immune checkpoint blockade treatment.

## Materials and Methods

### Human Tissue Samples Collection

All human samples used in this study were collected from 36 patients who were subjected to clinical surgery in the First affiliated hospital of Bengbu medical college (Bengbu cohort). Before RNA isolation and protein extraction, samples were stored at -80°C.

### Immunohistochemistry

Target tissues were cut to 4 µm thick, then deparaffinized, and rehydrated with xylene and graded alcohols (from 100% to 70%). After antigen retrieval with five mM citrate buffer, 3% H_2_O_2_ was used to inactivate endogenous peroxidase. The sections were blocked with goat serum for 30 min at room temperature and incubated with primary antibodies overnight at four°C. The sections were washed with phosphate-buffered saline (PBS) three times and incubated with a biotinylated secondary antibody at room temperature for two h. Diaminobenzidine was used as a chromogen substrate. Finally, the sections were counterstained with hematoxylin. Antibody information was listed in **Table S9**.

### Data Collection and Preprocessing

We methodically collected the molecular and clinical data of 1080 breast cancer patients from The Cancer Genome Atlas (TCGA) database, Molecular Taxonomy of Breast Cancer International Consortium (METABRIC) database, and Gene Expression Omnibus (GEO) database. Patients without survival information were excluded from further evaluation. Detailed methods are provided in the **Supplementary Methods**.

### Consensus Clustering Based on Representative Immune Signatures

The 83 signatures that are known to be associated with immune activity in tumor tissue collected from earlier study (6). To comprehensively illustrate the characteristics of 83 immune signatures, we superimposed proteins within each immune signature onto the protein-protein interaction (PPI) network based on the STRING database (http://string-db.org/) (14). We performed univariate Cox regression analysis to determine its prognostic value in the TCGA-BRCA (Breast invasive carcinoma) and METABRIC cohorts using the survminer package in R.

Before clustering, we discovered potential representative signatures based on weighted gene correlation network analysis (WGCNA) (15). First, single-sample gene set enrichment (ssGSEA) analysis (16) was performed across all 1080 BRCA patients to generate the gene set scores of each immune signature, as implemented in the GSVA package in R (17). Spearman correlation coefficients were computed among each immune signature to create a correlation matrix. Next, the correlation matrix was subject to a specified power and clustered using the WGCNA package in R with the following parameters: power = 20, TOMType = “signed”, pamStage = F, and minModuleSize = 3 (6). In every confirmed module, the “Eigen-signature” was defined as the potential representative immune signature, which indicated the highest connectivity in each module. We then obtained six potential representative immune signatures from the WGCNA that were further validated by the strategy put forth in “cluster validation by predictive strength” (18). The mclust package in R (19) was employed to perform model-based clustering using finite normal mixture modelling. This approach revealed one potential signature that lacked robustness, and it was excluded from further analysis. Therefore, we ultimately obtained five representative immune signatures, each of which are represented by one of the five signature similarity modules. The maximal Bayesian information criterion (BIC) was leveraged to identify subtype patterns and classify patients by testing from 2 to 28. The number of clusters was further validated by tSNE (20) to measure the robustness. Finally, we classified TCGA-BRCA patients into six clusters based on the final five representative immune signatures for further analyses.

### Establishment of the Immune Biological Signature

To explore the immune-associated patterns of specific tumors, we established a scoring system, termed the immune biological signature, to assess the immunopatterns of individual patients with breast cancer. The composition of the immune biological signature was investigated as follows.

Supervised analysis was performed to identify the subtype-specific genes that were differentially expressed among the established immune subtypes. To evaluate the predictive power of the differentially expressed genes, we randomly classified TCGA-BRCA samples into training and testing sets at a ratio of 7 to 3. Three well-established machine learning algorithms adapted from Yuan et al. (21), logistic regression (LR), support vector machine (SVM) (22) and random forest (RF) (23), were used to predict the subtypes (as a binary variable) using the least absolute shrinkage and selection operator (LASSO) penalty (24) to filter the informative genomic features. The performance of distinct classifiers was assessed using the fivefold cross-validation method: each training set was randomly divided into five sections. LASSO and model selection were applied based on 4/5 of the training set, and model prediction was applied based on 1/5 of the training set. The predictions of five iterations were registered and visualized with receiver operating characteristic (ROC) curves. Since the AUC score of the RF algorithm surpassed that of the two other algorithms, we performed LASSO and model selection on the whole training set using RF. We evaluated its predictive power across the entire testing set. The biological value of all subtype-specific genes was analyzed using a univariate Cox regression model. We then performed principal component analysis (PCA) to generate an immune biological signature and scored the signature using principal component 1 (PC1). The advantage of this method is that the score is concentrated on the set with the well correlated (or anticorrelated) gene block while reducing the weight of the contribution of genes that were not associated with other set members. Finally, we defined the immunotherapeutic benefit (ITBscore) of each patient using the method adapted from GGI and the TMEscore (25, 26):


$$\text{ITBscore}=\Sigma {\text{ PC}1}_{\text{i}}-\Sigma {\text{ PC}1}_{\text{j}}$$

where i is the gene whose Cox coefficient is positive and j is the gene whose Cox coefficient is negative.

### Statistical Analysis

Student’s t-test and the Wilcoxon rank-sum test were utilized to compare normally distributed variables and non-normally distributed variables, respectively. For comparisons of more than two groups, the Kruskal-Wallis test was used. Two-sided Fisher exact tests were used to analyse contingency tables. Correlations were assessed with Spearman or Pearson correlation analyses as stated in the text. Survival analysis was conducted using the Kaplan-Meier method with the log-rank test; each set’s cut-off point was evaluated using the survminer package in R. ROC curves were generated using the pROC package (27) and used to assess the predictive ability of TMB, the ITBscore, and their combination. The likelihood ratio test was applied to compare the AUCs using the lmtest package in R. All heatmaps were generated using the ComplexHeatmap package in R (28). The p values were two-sided and adjusted according to the Benjamini–Hochberg approach (BH) to control for the false discovery rate (FDR). Genes with an FDR < 0.05 were considered significant. All analyses and images were conducted and generated using R programming language (29) unless indicated.

## Results

### The Landscape of Immune Subtypes in Breast Cancer

**Figure 1:**
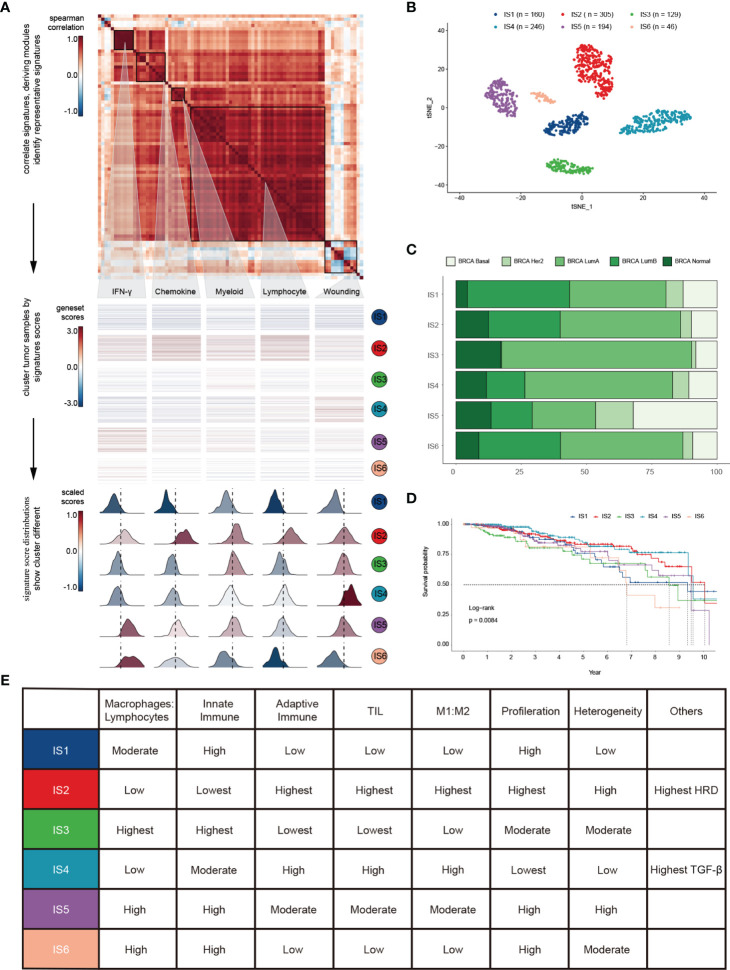
Landscape of immune subtypes in breast cancer. **(A)** Module distribution and cluster results. Top: Spearman correlation coefficients of 83 immune gene signatures. Boxes display five modules with shared associations. Middle: Six immune subtypes clustered by five representative signatures. Bottom: Distributions of signature scores within the six subtypes, with a dashed line indicating the median. **(B)** t-SNE plot showing the different immune subtypes. **(C)** Survival analysis grouped by immune subtype. **(D)** Distribution of immune subtypes and TCGA subtypes. **(E)** Summary of crucial characteristics by immune subtype.

To depict immune response characteristics unique to breast cancer, explore the rationality of immune phenotypes, and estimate the impact from the tumor microenvironment (TME), we performed a comprehensive immunogenomic analysis using the multi-omics profile of 1080 TCGA-BRCA samples. To better illustrate the features of 83 immune-associated signatures utilized to distinguish the immune phenotypes, we constructed a protein-protein interaction (PPI) network based on the STRING database (**Figures S1A, B**) and evaluated its prognostic value (**Figure S1C** and **Table S1**). By scoring 83 signatures and applying the cluster method, we identified five representative immune signatures (**Figure 1A**): “Module3_IFN_score”, representing the IFN-gamma response (abbreviated “IFN-γ” in the text and figures) (30), STAT1_19272155, illustrating chemokine signalling (abbreviated “Chemokine” in the text and figures) (31), G_SIGLEC9, representing myeloid cell activation involved in immune responses (abbreviated “Myeloid” the in text and figures) (32), HER2_Immune_PCA_18006808, representing the regulation of lymphocyte activation (abbreviated “Lymphocyte” in the text and figures) (33), and Troester_WoundSig_19887484, representing the negative regulation of angiogenesis (abbreviated “Wounding” in the text and figures) (34). Thus, we classified breast cancer patients into six clusters, termed immune subtypes (**Figures S1D, E**): IS1-IS6 (160, 305, 129, 246, 194, and 46 patients, respectively). The clusters were further validated using t-distributed stochastic neighbor embedding (tSNE), which assured the accuracy of consensus clustering (**Figure 1B**).

IS1 (defined as immunologically quiet) was characterized by a low proportion of TILs within the TME, low M1/M2 macrophage polarization, a high proliferation rate (**Figure S1F**). IS2 was characterized by chemokine dominant and had the highest proportion of TILs, indicating the highest adaptive immune response and lowest innate immune response. IS2 was dominated by M1 macrophages and had the highest proliferation rate, with the highest homologous recombination deficiency (HRD) (**Figure S1F**). IS3 (described as lymphocyte depleted) was characterized by an extreme lack of lymphocytes in the TME and exhibited the lowest adaptive immune response and highest innate immune response contrary to IS2 (**Figure S1F**); it was dominated by M2 macrophages and mainly consisted of breast invasive carcinoma luminal A rather than luminal B (**Figure 1C**). IS4 (wounding dominant) was defined by the lowest expression levels of wound healing signatures, suggesting weak proliferation ability, elevated TILs, high M1/M2 macrophage polarization, few macrophages/lymphocytes (**Figure S1F**), and a strong TGF-β signal. IS4, along with IS2, displayed better survival benefits than other subtypes (**Figure 1D**). IS5 (innate immune dominant) showed a dominant natural immune response signature, high proliferation rate, heterogeneity (**Figure S1F**), and the highest proportion of the basal subtype (**Figure 1C**), with a high M2 response. IS6 (IFN-γ dominant) displayed a high proportion of macrophages and dendritic cells, and a low proportion of TILs with an even distribution of CD4 and CD8 T cells (**Figure S1F** and **Figure 3A**). Finally, the distinct TME and principal features of tumor samples over the six immune subtypes were summarized (**Figure 1E** and **Figure S1F**; **Table S2**).

### Transcriptomic Characteristics of Immune Subtypes in Breast Cancer

**Figure 2:**
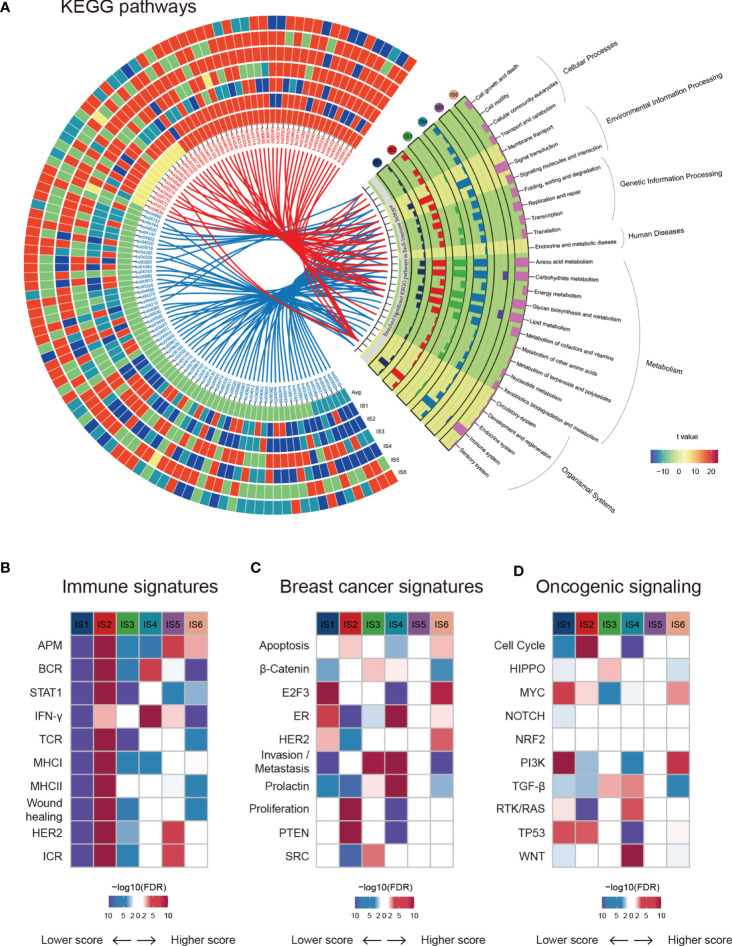
Functional annotation of immune subtypes. **(A)** KEGG functional categories and pathways of each immune subtype. **(B–D)** ssGSEA was applied to test the significance of differentially expressed genes in the immune-associated signatures, breast cancer signatures, and oncogenic pathways in specific immune subtypes compared with others.

Gene Ontology (GO) and Kyoto Encyclopedia of Genes and Genomes (KEGG) pathway analyses highlighted the transcriptomic characteristics of immune subtypes, and a total of 115 significantly (Kruskal-Wallis test, BH-adjusted p < 0.05) enriched pathways (**Table S3**) and six GO categories (**Figure S2**) were identified. A Circos plot was used to display the KEGG pathway landscape and dominant functional categories (**Figure 2A**). The DIDS package in R was utilized to calculate the differential abundance of a specific subtype against the remainder of the samples (**Figure 2A**, left), and all significant pathways of each subtype (Wilcoxon test, BH-adjusted p < 0.05) were aggregated to KEGG orthology (KO) functional categories (**Figure 2A**, right). From the graph above, we can see that the “Genetic information processing” and “Metabolism” functional categories were enriched in all subtypes except for IS5, which showed a faint distinction compared with the others. Moreover, the chemokine signalling pathway (ko04062) in the immune system was the most significantly enriched (BH-adjusted p < 1.0 × 10^-72^) among the six subtypes. In summary, their roles in the anti-tumor immune response within the TME are worthy of further research.

Next, we estimated some specific functional signatures across the immune subtypes. As expected, the immune signatures were strongly associated with the immune subtypes (**Figure 2B**). IS1 and IS3 were characterized by a lower immune signature score, whereas IS2 was characterized by a higher adaptive immune response score (**Figures 1D** and **Figure 2B**). IS3 and IS4 were characterized by elevated β-catenin and invasion signatures, indicating increased metastasis ability (**Figure 2C**). It is worth mentioning that IS5 was not significantly correlated with breast cancer signatures or oncogenic signalling pathways (**Figures 2C, D**). Analogously, NRF2 signalling pathways showed no change across immune subtypes (**Figure 2D**). In summary, breast cancers with different immune subtypes displayed different mRNA signatures.

### Extrinsic Immune Escape Mechanisms of Breast Cancer

To dissect the molecular mechanisms and explore the distinct characteristics of immune escape in different immune subtypes, we comprehensively investigated the relevant factors from the TME. In light of the immunoediting theory (35), extrinsic escape mechanisms may arise from a lack of innate immune sensing (such as activation of the WNT–β-catenin signalling pathway and inhibition of the cGAS–STING pathway) (36), inhibited infiltration of immune cells (such as Batf3-expressing dendritic cells (DCs) and CD8^+^ T cells) (37, 38), formation of an immunosuppressive state (such as recruiting Tregs and myeloid-derived suppressor cells (MDSCs), production of the immunosuppressive cytokines VEGF and TGF-β) (39), and other alterations in the TME (36, 40).

**Figure 3:**
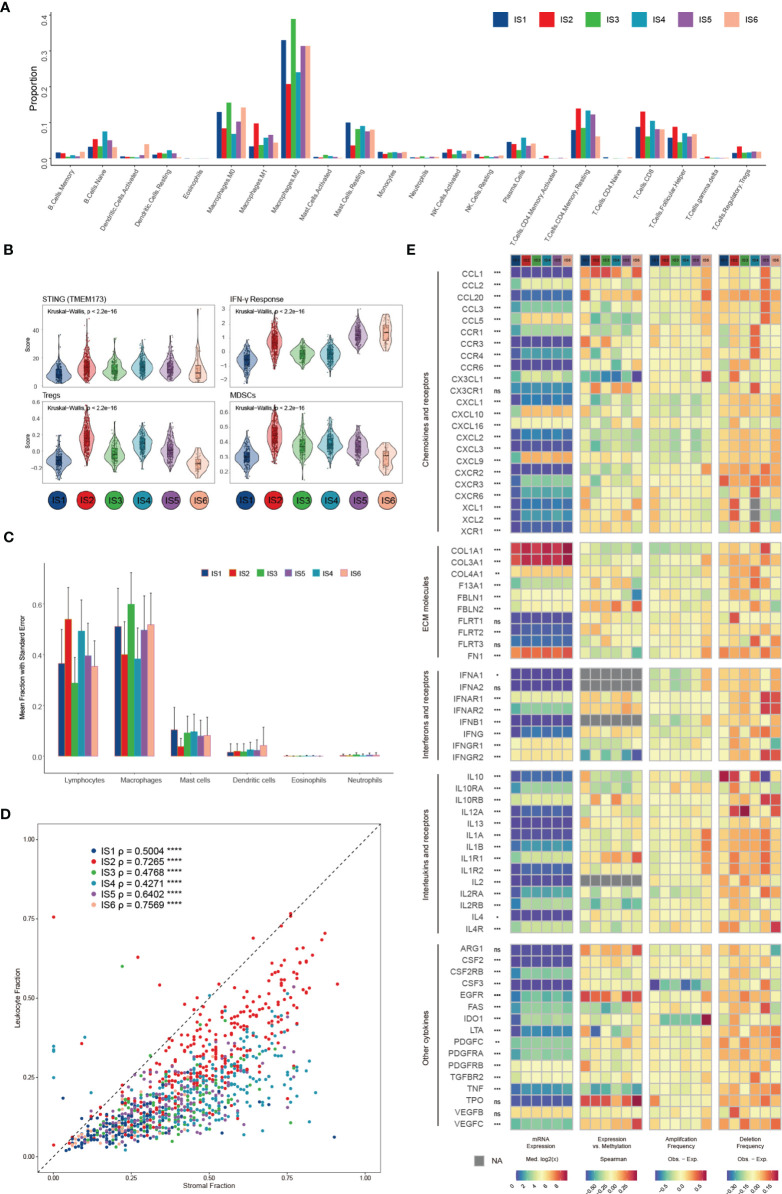
Potential extrinsic regulation of immune escape in breast cancer. **(A)** Proportions of 22 immune cell types within the TME according to the immune subtype **(B)** Mean trend scores of MDSCs, Tregs, the IFN-γ response, and mRNA expression of STING among subtypes. **(C)** The proportions of main categories of immune cells estimated by CIBERSORT according to distinct immune subtypes. **(D)** Leukocyte proportions (ρ=LF/SF) of distinct immune subtypes **(E)** From left to right: mRNA expression (median normalized expression levels), expression versus methylation (gene expression correlation with DNA methylation beta-value), amplification frequency, and deletion frequency for regulators.

We compared the approximate proportions of different immune cells over six subtypes in the TME (**Figure 3A**). The interaction network displayed the connections between tumor-infiltrating immune cells and their prognostic value in breast cancer patients’ overall survival (OS) (**Figure S3A**). IS2 possessed the largest fraction of Tregs and MDSCs (**Figure 3B**), which might lead to a strong immunosuppressive effect even though it had more lymphocytes (**Figure 3C**) (35). IS3 acquired more innate immune cells than others, suggesting the ability of cancer immunosurveillance. The expression of STING (**Figure 3B**), which is critical for the spontaneous initiation of innate immunity, and other proteins potentially related to natural innate immunity among the six subtypes also supported this hypothesis (**Figure S3B**). In addition to immune cells, stromal cells also play an essential role in the tumor immune response. There were more stromal cells, such as fibroblasts, endothelial cells, and chondrocytes, in IS4 than in the other subgroups (**Figure S3C**). Fibroblasts, specifically cancer-associated fibroblasts (CAFs), can inhibit the infiltration of immune cells, especially CD8^+^ T cells and natural killer (NK) cells, into the TME and suppress their functions within the tumor (41, 42). Moreover, the low leukocyte fraction (LF)/stromal fraction (SF) ratio in IS3 and IS4 may result in increased tumor proliferation and cancer stemness and promote invasion and metastasis (**Figure 3D**) (42).

Finally, we explored the gene expression of chemokines, ECM proteins, interleukins (ILs), IFNs, and other vital cytokines and their receptors (**Figure 3E**), conceivably indicative of their functions in shaping the TME, across each subtype. Cytokines with the most variation between subtypes included CXCL10 and PDGFRB (**Figures S3D–F**). The DNA methylation of some cytokine genes, such as TNF and LTA, negatively correlates with gene expression, indicating epigenetic silencing (**Figure S3G**). One hundred seventy-one miRNAs were identified as potential extrinsic cytokine regulators within the TME (**Figure S3H**). In general, the observed difference in each subtype in the TME might have implications in regulating immune escape and highlight the biological importance of breast cancer.

### Intrinsic Immune Escape Mechanisms of Breast Cancer

In addition to the influence of the TME, tumor cells can also evade the anti-tumor immune response through intrinsic variations. The innate mechanisms of immune escape are associated with reduced immunogenicity (36), increased resistance to the cytotoxic effects of immunity, and alterations in the expression of immune checkpoint proteins (35).

**Figure 4:**
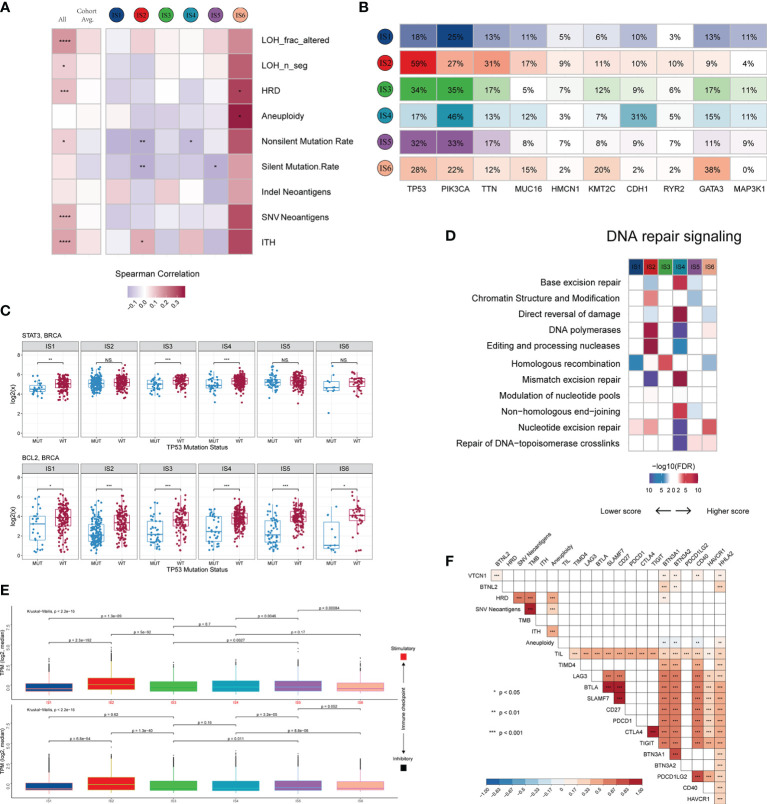
Potential intrinsic regulation and genomic alterations in breast cancer. **(A)** Correlation between DNA damage and immune infiltrates. From left to right: all BRCA samples; averaged by immune subtype; grouped by immune subtype. **(B)** Top 10 genes with the highest mutation frequency across immune subtypes. **(C)** Gene expression patterns of STAT3 and BCL2 grouped by TP53 mutation. **(D)** Difference in DNA repair signalling pathways. **(E)** Median expression levels of 24 co-inhibitory and 37 co-stimulatory genes across immune subtypes. **(F)** Correlation of immunomodulators and tumor immunogenicity indicators. *P < 0.05; **P < 0.01; ***P < 0.001; ns, not significant.

We investigated not only the potential elements associated with tumor antigens, such as aneuploidy, homologous recombination deficiency (HRD), neoantigens, and intratumoral heterogeneity (ITH) (**Figure S4A**), but also the relationship between DNA damage and immune infiltrates (**Figure 4A** and **Figure S4B**). LF showed a positive correlation with HRD, aneuploidy, and ITH, with the strongest correlation observed in IS6 and IS2, and a negative correlation with mutation load, particularly in IS2, IS4 and IS5. These results suggest a differential effect of multiple smaller, focal copy number events versus more significant immune infiltration events in specific immune subtypes. We further explored the genomic alterations associated with subtypes (**Figure 4B** and **Table S4**). IS1, IS3, IS4, and IS5 were mainly enriched in mutations in PIK3CA (25%, 35%, 46%, and 33%, respectively), one of the most mutated genes in solid cancers (43). IS6 was mainly distinguished by an enrichment of mutations in GATA3, which disrupts epithelial-to-mesenchymal transition (EMT) and inhibits the tumor-initiating ability of luminal progenitor cells and metastasis in breast cancer (44). In IS2, 59% of samples had a mutation in TP53, which affected the expression of STAT3 and BCL2 (**Figure 4C** and **Figure S4C**), and participated in resistance to cytotoxic effects by inducing anti-apoptotic mechanisms. Because of the higher TP53 mutation rate and TMB in IS2 (**Figure S4D**), we explored the mutational signature of IS2 by performing Bayesian non-negative matrix factorization (Bayesian NMF) analysis (45) of the mutations stratified by 96 trinucleotide contexts. Bayesian NMF analysis revealed four distinct patterns of mutagenesis operating among 33251 single nucleotide variants (SNVs) in IS2 (**Figure S4E**). The four mutation signatures were “APOBEC”, “DNA MMR deficiency”, “BRCA1/2 mutations” and “Pol ϵ mutations” (31.69% SNVs, 20.39% SNVs, 27.56% SNVs and 20.34% SNVs, respectively). We finally examined the 11 DNA repair signalling pathways curated by the Wood laboratory (46) to monitor the ability to protect the integrity of the genome (**Figure 4D**). Overall, these analyses showed distinct characteristics of genomic variations according to the immune subtype.

Immune checkpoint molecules are critical for immune escape and cancer immunotherapy. To advance these studies, we surveyed the expression patterns of immunomodulators at the mRNA, methylation, and somatic copy number alteration (SCNA) levels. Active immune checkpoint molecules are referred to as co-stimulatory and co-inhibitory molecules (https://www.rndsystems.com/cn/research-area/co–stimulatory-and-co–inhibitory-molecules) and may be indicative of their role in shaping the TME. As shown in **Figure S4F**, the expression of immunomodulators was lower in IS1 than in the other subtypes. The immunomodulators with the most remarkable variations between subtypes included SLMF6 (BH-p adjusted < 1.0 × 10^-72^) and CTLA4 (BH-p adjusted < 1.0 × 10^-71^), both of which were highly expressed in IS5 and weakly expressed in IS1. Specifically, the median transcript per millions (TPMs) of 24 inhibitory and 37 stimulatory immunomodulators were similar between subgroups IS1, IS3, IS4, and IS5 and significantly lower than IS2 but higher than IS6 (**Figure 4E**). Moreover, we calculated the Spearman correlation coefficients between immunomodulators and aneuploidy, neoantigens, TMB, HRD, TILs, and ITH. The results indicated that the expression levels of most immunomodulators showed positive correlations, whereas aneuploidy did not seem to be associated with BTN3A1, BTN3A2 and CD40 (**Figure 4F**).

### Clinical Characteristics and Biological Traits of the Immune Biological Signature

**Figure 5:**
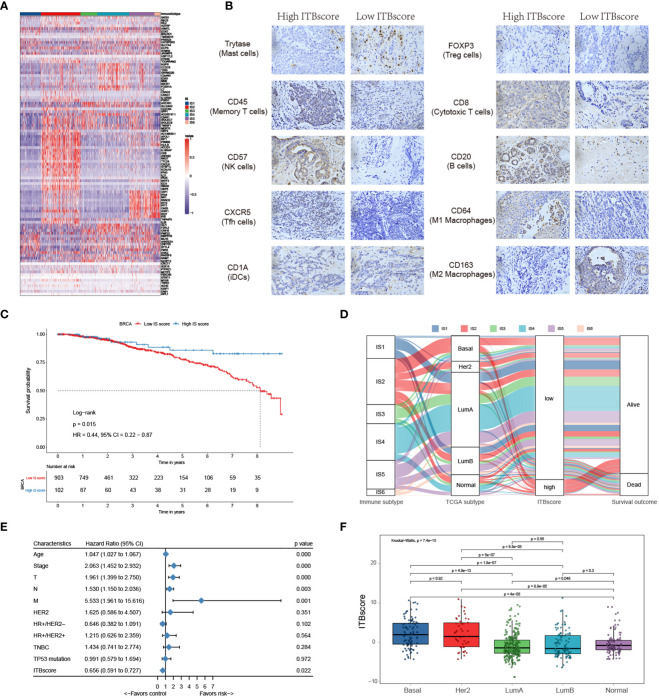
Construction of the immune biological signature. **(A)** DEGs were used to construct the immune biological signature. **(B)** Representative immunohistochemical images of infiltrated immune cells in Bengbu cohort between high- and low- ITBscore groups. **(C)** Kaplan-Meier curves for patients in the BRCA cohort divided into high and low ITBscore subgroups. **(D)** Alluvial diagram indicating immune subtypes in groups with different BRCA subtypes (basal, Her2, LumA, LumB, and normal), ITBscores, and survival outcomes. **(E)** Prognostic value of the ITBscore and classic clinicopathological covariates in the high/low ITBscore subgroups. **(F)** Distribution of the ITBscore among TCGA-BRCA molecular subtypes.

As shown by the above results, distinct immune subtypes present different immune and biological characteristics. The differential transcriptome of each immune subtype contributed to these consequences used to construct the immune biological signature. To broadly evaluate the patterns of immune subtypes in shaping different TME landscapes and genomic states, we established a scoring system to quantify the immune biological signature by applying a rigorous machine learning method (**Figure S5A**) termed the ITBscore. We also assessed the power of differentially expressed genes (DEGs) in classifying distinct immune subtypes (**Figure 5A**). Next, we explored the immune biological signature’s molecular mechanism and questioned whether the ITBscore could predict a patient’s outcome to immunotherapy. The 1080 patients in the TCGA-BRCA cohort were assigned to high and low ITBscore subgroups according to the cut-off value acquired using the survminer package in R. Patients in the high ITBscore group displayed a significant survival benefit compared with those in the low ITBscore group, and the 10-year survival rates were 80.7% and 41.2%, respectively (**Figure 5C**). The trait changes in each patient according to the clusters were visualized using an alluvial diagram (**Figure 5D**).

Furthermore, we performed univariate Cox regression analysis to determine whether the ITBscore could be an independent prognostic biomarker for breast cancer. To this end, the ITBscore and other characteristics of breast cancer patients, including age, clinical stage, TNM status, histological type, Her2 status and TP53 status, were examined. As shown in **Figure 5E**, the results indicated that the ITBscore is a robust and independent prognostic biomarker for estimating breast cancer patient outcomes (HR: 0.656, 95% CI: 0.591-0.727), and the correlation between the molecular subtype and ITBscore was significant (**Figure 5F**). The basal and Her2 subtypes, which are associated with more favorable prognoses in response to immunotherapy (47), presented notably higher ITBscores than the other three subtypes (Kruskal−Wallis, p = 7.4 × 10^−15^).

One factor associated with a good prognosis in the high ITBscore group might be the relatively high proportion of TILs. Patients with a high ITBscore had significantly larger proportion of T helper cells, CD8 T cells, and activated/resting CD4 memory T cells, with considerably fewer M2 macrophages and mast cells than patients with a low ITBscore (**Figure 5B**). The correlations of TILs in the high and low ITBscore groups are shown in **Figure S5B**. The next section of the study focused on molecular traits and genomic alterations. By employing gene set enrichment analysis (GSEA) of the whole transcriptome between the two groups, we discovered that immune response-associated gene sets, such as the inflammatory response, IL6-JAK-STAT3 signalling, and interferon α/γ response, were active in the high ITBscore group. However, some gene sets, including adipogenesis, fatty acid metabolism, and oxidative phosphorylation, were inhibited in the low ITBscore group (**Figures S5C, D**), consistent with the findings that patients from the high ITBscore group demonstrated better survival than those from the low ITBscore group. Therefore, the clinical benefits of the high ITBscore group might be associated with the enhanced G2/M checkpoint. We further explored the association between the ITBscore and mutation patterns using a random forest algorithm with two thousand iterations in the Boruta package in R and confirmed 14 significantly variant genes (**Figures S5E, F**). Preclinical (48) and clinical (49) studies have reported the influence of altered genes on immune checkpoint blockade. Only a few of these genes are associated with sensitivity or resistance in BRCA, such as TP53 and CDH1. These results show that an immune biological signature could be employed to estimate patients’ clinical characteristics and provide an original perspective for investigating immune-associated somatic mutations that shape the TME and function in immune checkpoint blockade therapy.

### Immune Biological Signature Predicts Immunotherapeutic Benefits

**Figure 6:**
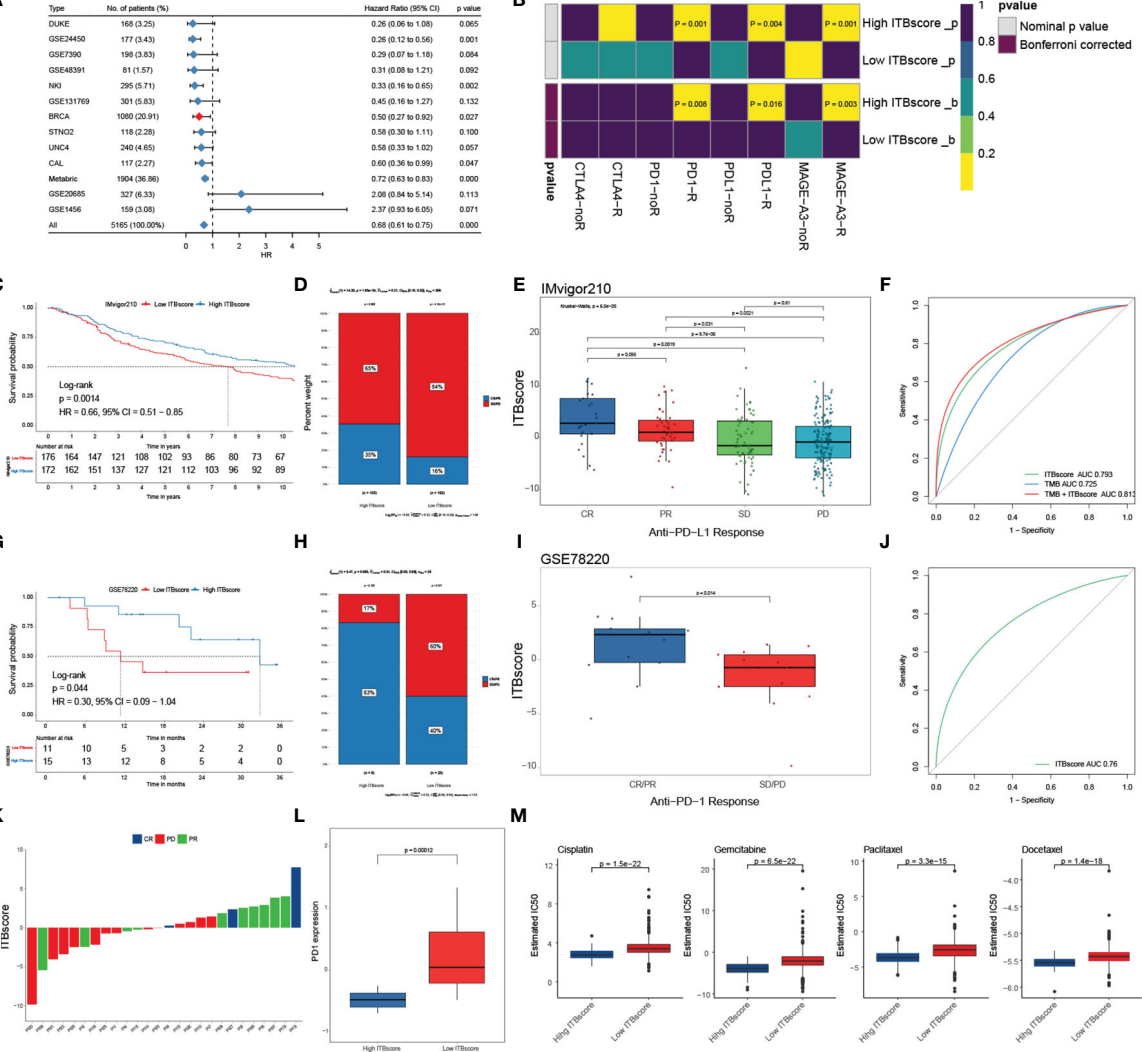
Role of the ITBscore in predicting immunotherapeutic prognosis. **(A)** The ITBscore was used to evaluate clinical prognosis in independent cohorts of breast cancer patients with high/low ITBscores. **(B)** Differential putative immunotherapeutic response in the high/low ITBscore subgroups. **(C)** Kaplan–Meier curves of the anti-PD-L1 response in the high and low ITBscore subgroups of patients in the IMvigor210 cohort. **(D)** The percentage of patients with high and low ITBscore groups with an anti-PD-L1 immunotherapeutic response. CR, complete response; PR, partial response; SD, stable disease; PD, progressive disease. **(E)** Distribution of the ITBscore in diverse anti–PD-L1 clinical response subtypes. **(F)** AUC value shows the predictive performance of the ITBscore and TMB in the IMvigor210 cohort. **(G)** Kaplan–Meier curves of the anti-PD-1 response in the high (n = 6) and low (n = 20) ITBscore subgroups in the GSE78220 cohort. **(H)** The percentage of patients with high and low ITBscore groups with an anti-PD-1 immunotherapeutic response. **(I)** Distribution of the ITBscore in diverse anti–PD-1 clinical response subtypes. **(J)** AUC value showing the predictive performance of the ITBscore in the GSE78220 cohort. **(K)** Distribution of the ITBscore in every patient with a clinical response to anti-PD-1. **(L)** Differences in PD1 in the high/low ITBscore subgroups. **(M)** Differential chemotherapeutic responses of the high/low ITBscore subgroups based on the IC50 value available in the GDSC database.

To further assess the accuracy of the ITBscore model, we exercised 12 independent breast cancer cohorts using the immune biological signature established with the TCGA-BRCA cohort to evaluate its clinical value (**Figure 6A** and **Figure S6A**). The value of the signature to predict OS up to ten years in the high and low ITBscore groups of all breast cancer datasets except for GSE1456 (HR: 2.37, 95% CI: 0.93 − 6.05) and GSE20685 (HR: 2.08, 95% CI: 0.84 − 5.14) was determined, as shown in **Table S5**. Furthermore, we performed a pan-cancer analysis of the prognostic value of the ITBscore in 32 independent TCGA cohorts involving 10140 patients (**Figure S6B**, detailed in **Table S6**). The ITBscore was identified as a robustly favorable prognostic biomarker in ten TCGA cohorts, albeit its value was heterogeneous in some cancers, such as adrenocortical carcinoma, colon adenocarcinoma, lower-grade glioma, and stomach adenocarcinoma.

Immune checkpoint inhibitors (ICIs), such as atezolizumab and avelumab, which target PD−L1, pembrolizumab and nivolumab, which target PD-1, and tremelimumab, which targets CTLA-4, have been applied as cancer immunotherapy during the past few decades (50–54). However, some of them were proven in solid tumors, including breast cancer, with finite efficacy (50). Next, we evaluated the predictive value of the ITBscore in response to immune checkpoint blockade. We first predicted the potency of immunotherapy for patients in the TCGA-BRCA cohort with high and low ITBscores using the TIDE and SubMap algorithms. We found that patients with a high ITBscore were more likely to respond to immune checkpoint blockade with anti-PD-1 (Bonferroni corrected p = 0.008), anti-PD-L1 (Bonferroni corrected p = 0.016), and anti-MAGE-A3 (Bonferroni corrected p = 0.003) drugs (**Figure 6B**). We further explored the predictive value of the ITBscore in the IMvigor210 (anti-PD-L1) (51) and GSE78220 (anti-PD-1) (52) cohorts. In both immunotherapy cohorts, patients in the high ITBscore group presented remarkably better survival and clinical benefits than those in the low ITBscore group (HR: 0.66, 95% CI: 0.51 − 0.85, IMvigor210, **Figure 6C**; HR: 0.30, 95% CI: 0.09 − 1.04, GSE78220, **Figure 6G**). The predictive value of the ITBscore was further investigated to determine the acquisition of therapeutic advantages for patients in the IMvigor210 (**Figures 6D–F**) and GSE78220 (**Figures 6H–K**) cohorts with a high ITBscore. Since a higher somatic tumor mutational burden (TMB) is correlated with better OS and improved survival in response to ICI immunotherapy in several cancers (55), we estimated the difference between TMB and the ITBscore in the IMvigor210 cohort using receiver operating characteristic (ROC) curve analysis (27). The ITBscore presented a greater predictive advantage than TMB (likelihood ratio test, p < 0.001, **Figure 6F**), and the combination of TMB and the ITBscore enhanced the predictive ability compared with TMB or the ITBscore alone (likelihood ratio test, combination versus ITBscore, p = 0.007; combination versus TMB, p < 0.001; **Figure 6F**). After we assessed the predictive value of the ITBscore for immunotherapy with anti-PD-1 (GSE78220) and anti-PD-L1 (IMvigor210), we analyzed the anti-MAGE-A3 (GSE35640, **Figures S6C, D**) (53) and anti-CTLA-4 (GSE63557, **Figures S6E, F**) (54) immunotherapy cohorts using the same ITBscore model. Patients with higher ITBscores obtained clinical benefits from ICIs (the ITBscores of patients treated with ICIs are summarized in **Table S7**). In addition, patients with low ITBscores demonstrated significantly higher expression of PD-1, which suggested a potential response to anti-PD-1/L1 immunotherapy (**Figure 6L**).

Since chemotherapy is the standard treatment against cancer, we assessed the response to anticancer drugs, such as cisplatin, gemcitabine, paclitaxel, and docetaxel, which are frequently used as first-line treatments in breast cancer, using the ITBscore. The predictive model was generated using the GDSC dataset (56) *via* Ridge regression with 10-fold cross-validation. We compared the IC_50_ values of four chemotherapeutic drugs between patients from the high and low ITBscore groups and observed that patients with higher ITBscores were more sensitive to chemotherapy than those with low ITBscores (p < 0.001 for cisplatin, gemcitabine, paclitaxel, and docetaxel) (**Figure 6M**). The survival advantage of patients in the TCGA-STAD cohort subjected to chemotherapy, regardless of the ITBscore, was higher than that of patients not subjected to chemotherapy (log-rank test, p < 0.001, **Figure S6G**). Finally, the distribution of the ITBscore among molecular subtypes and histologic grades was assessed (**Figures S6H, I**). We found that the ITBsocre of CIN > MSI > GS > EBV existed such that, and the reduced ITBscore in the TNM stage. In summary, these results show that the ITBscore model generated from the immune biological signature is a robust biomarker and can predict an excellent response to immunotherapy in breast cancer patients.

## Discussion

Since it became clear that the expression of tumor-specific and tumor-selective antigens is affected by genetic alterations and epigenetic dysregulation in cancer cells, the feasibility of utilizing the immune response to anti-tumor therapy has been gradually acknowledged. Currently, antibody-mediated blockade of the PD-1/CTLA-4 pathways is an effective way to establish sustained immune responses and treat multiple cancers, including breast cancer, in clinical practice and ongoing trials (52, 53). However, the clinical response and survival benefits are usually restricted to a subset of patients. A complete understanding of the TME and an effective stratification approach are necessary. In the present study, we clustered patients with breast cancer into six immune subtypes inspired by the excellent work of Vésteinn Thorsson et al. (6). We detailed the immune content of patients in the TCGA-BRCA cohort using multiple methods. These analyses covered functional enrichment, potential extrinsic immune escape mechanisms (e.g., the accurate estimation of tumor-infiltrating lymphocytes (TILs) composition from the deconvolution of gene expression), and the intrinsic regulation of genomic homeostasis (e.g., an association between DNA damage and somatic alterations with immune infiltrates). All immune contents were compared among the six immune subtypes. Finally, we established a scoring system to predict the response of breast cancer patients to immunotherapy.

A different pattern of immune activation presents unique survival benefits. The IS3 and IS6 subtypes conferred the most adverse outcomes and displayed an immunosuppressed TME dominated by macrophages, lower lymphocytic infiltrates (high macrophages: lymphocytes) and a higher M2 macrophage content. By comparison, patients in the IS2 and IS4 subgroups had the most favorable prognosis, which may be associated with the role of the adaptive immune response in prevention of cancer progression (57). Furthermore, IS2 and IS4 were associated with higher M1 macrophage and lower M2 macrophage contents than IS3 and IS6, in agreement with several studies confirming that tumor-associated macrophages (TAMs) play intricate roles in tumor development (M1 phenotype for tumor prevention and M2 phenotype for tumor promotion) (58). IS2 was chemokine dominant and showed a more favorable survival benefit (likely by possessing the highest content of lymphocyte infiltrates and M1 content, indicating a vigorous anti-tumor immune response). However, IS2 also showed the most striking signatures of Tregs and MDSCs, two dominant immunosuppressive leukocytes that prohibit host defense anti-tumor responses. This inconsistency may be due to the difference of tumor aggressive behaviour between IS2 and other subtypes. Both the proliferation signature and ITH were the highest in the IS2 subtype, resulting in a relatively high growth rate beyond the control of the immune response. Another possible explanation for this finding is that tumor cells in IS2 have been remodeled by existing robust type I infiltrates that elude immune recognition. Although interferon-mediated viral sensing and antigen genes are actively engaged in survival benefits, interferon signatures without elevated antigen presentation exhibit an inverse relationship. Cancer cells that have been immune edited are characterized by the lack of genes related to antigen processing and production. In contrast to IS2, which is most likely controlled by immune editing, IS4 is most likely controlled immunologically, that is, *via* immune equilibrium. Chemokine signalling is involved in the differentiation of immune cells and may be a resistance mechanism to checkpoint inhibitors (59). For example, some studies have proven that some chemokines, especially CXCL9, CXCL10, CXCL11 and CXCR3, may improve chemotherapy by activating the paracrine axis and inhibiting the autocrine axis (59). Compounds that enhance the expression of CXCL9, CXCL10 or CXCL11 and decrease the expression of CXCR3 on tumor cells have displayed anti-tumor activity (60–62). Hence, drugs that target chemokine signalling may be practical to patients with the IS3 and IS5 subtypes rather than those with other subtypes. Production of IFN-γ is pivotal in determining the effectiveness of the immune response to pathogens (63). Previous research has focused on the production of IFN-γ by T cells and natural killer (NK) cells, but some data has observed the production of IFN-γ by antigen-presenting cells (APCs), such as macrophages and dendritic cells (64, 65). Adding to our results that IS6 is IFN-γ dominant with a high proportion of macrophages and dendritic cells but displayed a low proportion of TIL, these results exhibited the special properties of IS6 for the APCs-derived IFN-γ.

Cellular DNA sustains incessant invasion from internal mutagenesis and environmental agents. The fact that somatic mutations may influence the immune response was considered. Genome instability generated by the most diverse DNA-damaging agents would provoke an immune response if it were not for the DNA repair pathway. For example, most DNA repair pathways were enriched in IS2 and IS4 but not in IS1 and IS3, implying that DNA repair may promote the innate and adaptive immune responses. Cardinal DNA repair items may make a vast difference in confining chronic inflammatory signalling (66). Driver mutations such as those in TP53 and PIK3CA may reshape the immune landscape *via* the generation of neoantigens by promoting chromosomal imbalance (49). Wild-type TP53 signalling has been correlated with the recruitment and activation of immune cells (34) and recruitment of NK cells (especially resting NK cells) into the TME (67). Further studies that take these factors into account will need to be conducted.

Despite the extensive use of immunotherapeutic strategies for cancer patients, the efficacy and accuracy of routine biomarkers are limited. Some studies have proven that the expression of PD1/PD-L1 and CTLA-4, microsatellite instability (MSI) status, and mutation load are not effective for predicting the immune response in different types of cancer (68–70). Here, the ITBscore was established from the immune biological signature to support some computational algorithms and designed to evaluate survival benefits and improve the predictive response of immune checkpoint treatments. Comprehensive analyses showed that the ITBscore was a prognostic biomarker in breast cancer and was higher in patients with aggressive molecular subtypes (basal and HER2+) and characterized by a high tumor grade and poor OS. Basal-like breast cancer (TNBC) has increased immunogenicity compared with other molecular subtypes, indicating immunotherapeutic preference (71). In addition, recent evidence has shown relatively high levels of TILs and PD-L1 expression in the majority of HER2+ breast cancer patients. These characteristics may contribute to the application of immunotherapeutic treatments in this subset of patients (72). These results further illustrated that our methodological analyses to assess the TME are a promising predictive biomarker to advance precision immunotherapy in breast cancer patients. We also determined that the ITBscore was correlated with gene mutations, especially TP53 mutations, which is in line with a previous study showing that patients with TP53 mutations displayed clinical benefits from ICIs (49). These findings might raise the possibility that the ITBscore can improve the accuracy of immunotherapy and encourage the use of the combined strategy of both immunotherapy and gene mutations. In brief, for clinical applications, the ITBscore could be employed to comprehensively assess the immune content and associated TME cell infiltration characteristics in individual patients, define the tumor immunophenotype and direct more effective medical practice. Therefore, we conclude that the ITBscore could be an independent biomarker to predict the response to adjuvant chemotherapy and different immune checkpoint blockades.

Taken together, this study set out to explore the characteristics of the TME infiltration patterns in breast cancer and provides several original insights into the anti-tumor immune response, which may facilitate the development of cancer immunotherapy strategies.

## Data Availability Statement

Publicly available datasets were analyzed in this study. This data can be found here: https://portal.gdc.cancer.gov/, https://www.ncbi.nlm.nih.gov/geo/ under the accession numbers GSE1456, GSE20685, GSE78220, GSE35640 and GSE63557.

## Ethics Statement

The studies involving human participants were reviewed and approved by Department of Pathophysiology, Bengbu Medical College. The patients/participants provided their written informed consent to participate in this study.

## Author Contributions

TW and XZ conceived and designed this work. TW, TL, JZ, and ZL integrated and analyzed the data. TW and BL developed the methodology. TW, MS, YL, YZ, SZ, and WH collected data. TW, XG, RG, LW, and DD provided administrative, technical, or material support. TW, XZ, and TL wrote and edited the manuscript. TW, XZ, and JZ reviewed the manuscript. All authors read and approved the final manuscript.

## Funding

This work was supported by the Education fund item of Anhui province under Grant KJ2020A0588.

## Conflict of Interest

The authors declare that the research was conducted in the absence of any commercial or financial relationships that could be construed as a potential conflict of interest.

## Publisher’s Note

All claims expressed in this article are solely those of the authors and do not necessarily represent those of their affiliated organizations, or those of the publisher, the editors and the reviewers. Any product that may be evaluated in this article, or claim that may be made by its manufacturer, is not guaranteed or endorsed by the publisher.
