# Corrigendum: Immunogenomic landscape in breast cancer reveals immunotherapeutically relevant gene signatures

**DOI:** 10.3389/fimmu.2023.1134847

**Published:** 2023-01-20

**Authors:** Tao Wang, Tianye Li, Baiqing Li, Jiahui Zhao, Zhi Li, Mingyi Sun, Yan Li, Yanjiao Zhao, Shidi Zhao, Weiguang He, Xiao Guo, Rongjing Ge, Lian Wang, Dushan Ding, Saisai Liu, Simin Min, Xiaonan Zhang

**Affiliations:** ^1^ College of Life and Health Sciences, Northeastern University, Shenyang, China; ^2^ Department of Immunology, Bengbu Medical College, Bengbu, China; ^3^ Department of Pathophysiology, Bengbu Medical College, Bengbu, China; ^4^ Department of Radiology, Tian Jin Fifth’s Central Hospital, Tianjin, China; ^5^ College of Pharmacy, Beihua University, Jilin, China

**Keywords:** immune subtype, tumor microenvironment, immune escape, immunotherapy, breast cancer

In the published article, there was an error in the IHC images of Figure 5B as published. Four repeat IHC images were displayed cursorily. The corrected Figure 5B and its caption “Construction of the immune biological signature. (A) DEGs were used to construct the immune biological signature. (B) Representative immunohistochemical images of infiltrated immune cells in Bengbu cohort between high- and low- ITBscore groups. (C) Kaplan-Meier curves for patients in the BRCA cohort divided into high and low ITBscore subgroups. (D) Alluvial diagram indicating immune subtypes in groups with different BRCA subtypes (basal, Her2, LumA, LumB, and normal), ITBscores, and survival outcomes. (E) Prognostic value of the ITBscore and classic clinicopathological covariates in the high/low ITBscore subgroups. (F) Distribution of the ITBscore among TCGA-BRCA molecular subtypes. “ appear below.

**Figure 1:**
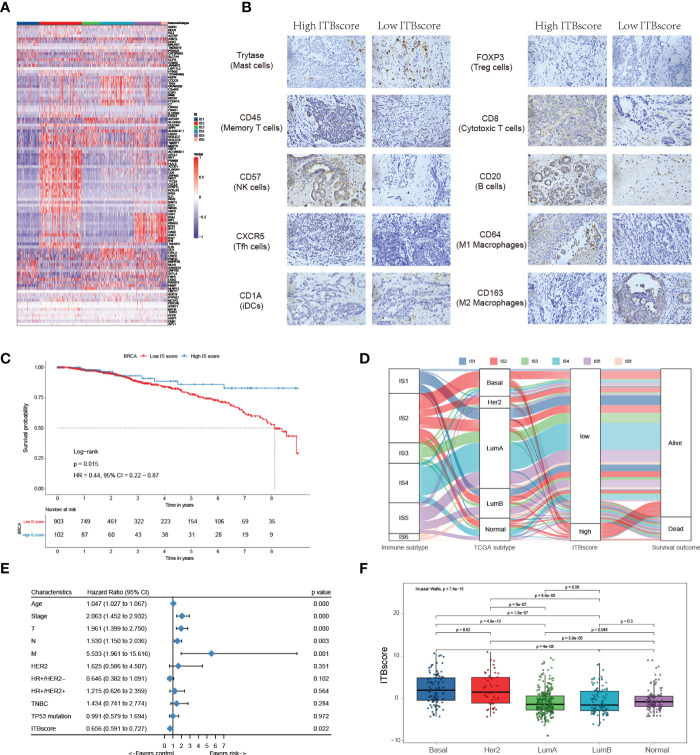
Construction of the immune biological signature. **(A)** DEGs were used to construct the immune biological signature. **(B)** Representative immunohistochemical images of infiltrated immune cells in Bengbu cohort between high- and low- ITBscore groups. **(C)** Kaplan-Meier curves for patients in the BRCA cohort divided into high and low ITBscore subgroups. **(D)** Alluvial diagram indicating immune subtypes in groups with different BRCA subtypes (basal, Her2, LumA, LumB, and normal), ITBscores, and survival outcomes. **(E)** Prognostic value of the ITBscore and classic clinicopathological covariates in the high/low ITBscore subgroups. **(F)** Distribution of the ITBscore among TCGA-BRCA molecular subtypes.

The authors apologize for this error and state that this does not change the scientific conclusions of the article in any way. The original article has been updated.

