# Supplementary material for: Immunogenomic Landscape in Breast Cancer Reveals Immunotherapeutically Relevant Gene Signatures
Source: Front Immunol. 2022 Jan 27;13:805184. doi: 10.3389/fimmu.2022.805184 (PMC8829007; doi:10.3389/fimmu.2022.805184)
Supplement: Supplementary file 1 [file DataSheet_1.pdf]

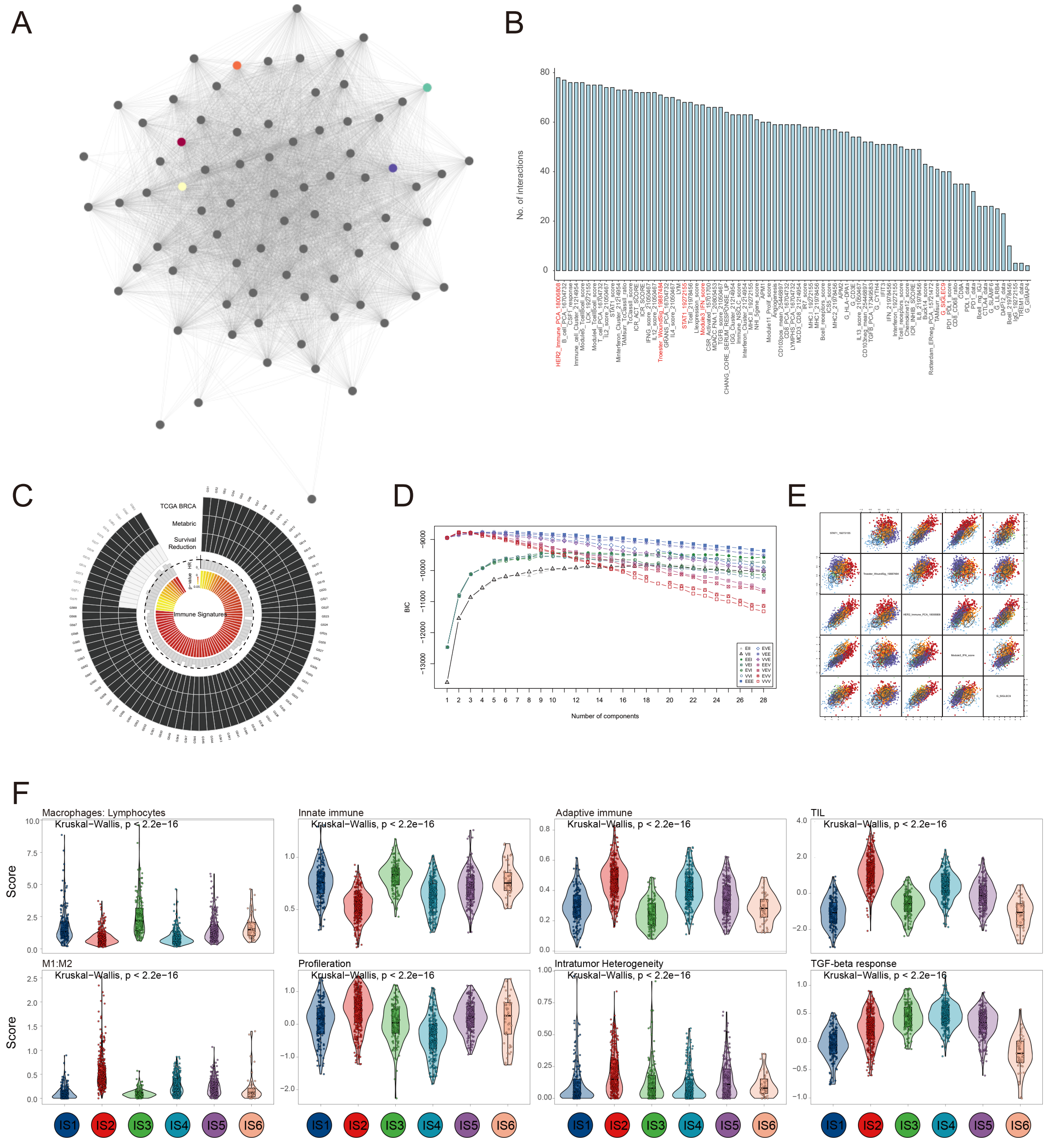

Fig S1. Identification and characterization of immune subtypes. (A) Allocation of 83 immune signatures in the PPI network. Nodes represent the 83 immune signature sets; edges connect locations that share PPIs. (B) Number of interactions among each immune gene set and superimposition of proteins in the PPI network are shown. The final five representative immune signatures are shown in red. (C) Circos plot correlating 83 immune signatures with prognostic status in the TCGA-BRCA and META-BRIC cohorts. HRs and p values are shown, and these immune signatures were associated with poor survival (D-E) Results from model-based clustering. (F) Scores of key immune characteristics grouped by immune subtype.

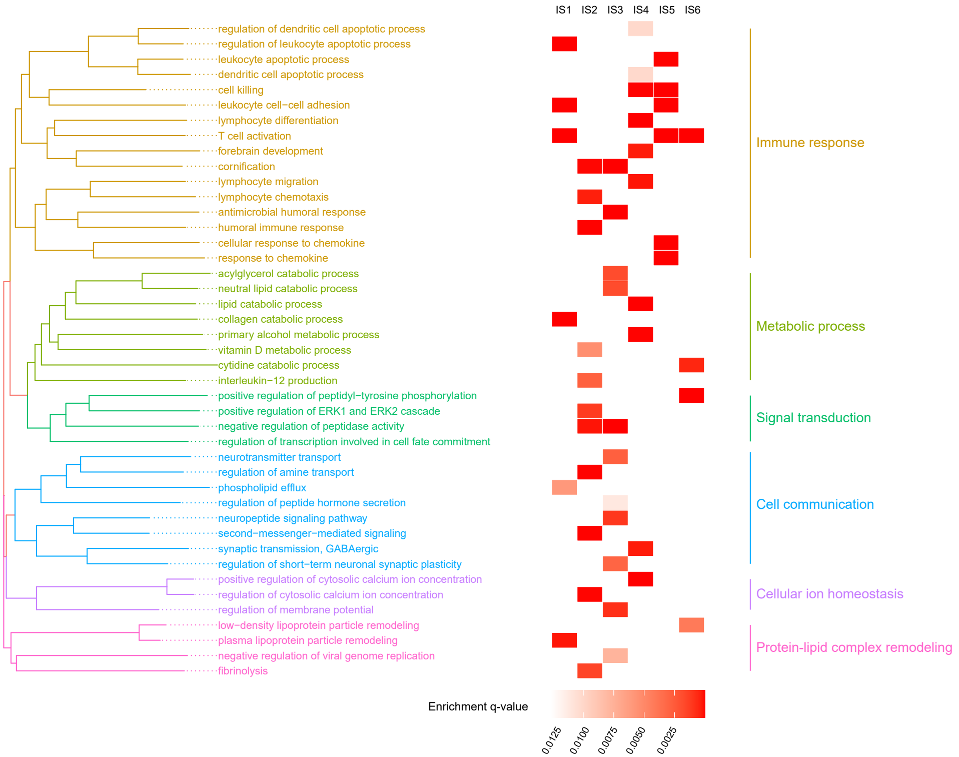

Fig S2. GO categories of DEGs in each immune subtypes are grouped according to functional theme.

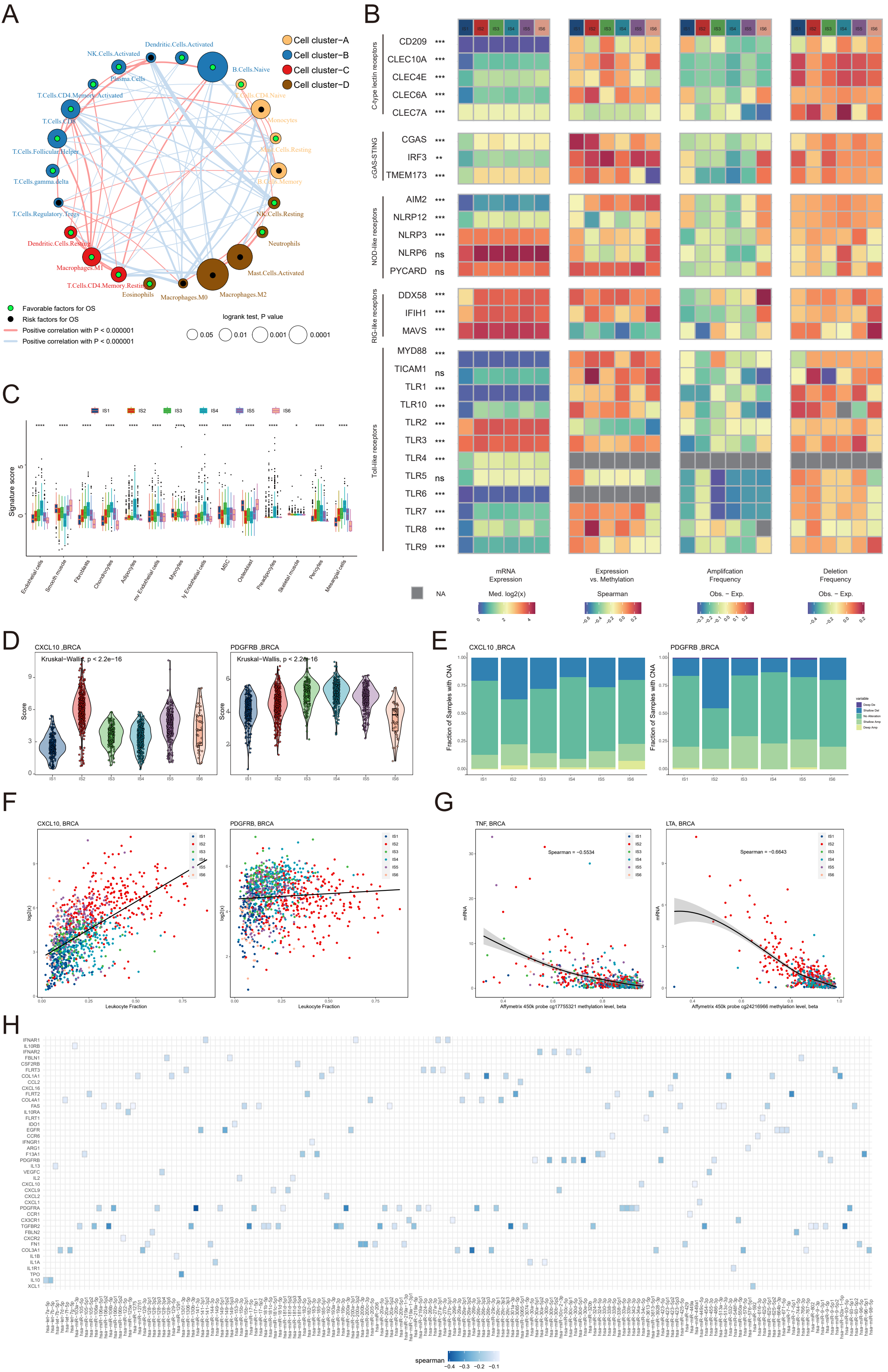

Fig S3. Cellular and molecular interactions within the TME. (A) Cellular associations of 22 immune cells in the TME. Points in green and black represent favourable factors and risk factors, respectively. The cell size indicates the p-value (log-rank test) for a distinct immune cell type to predict overall survival (OS). The thickness of the lines indicates the Spearman correlation value, with red representing a positive correlation and blue representing a negative correlation. (B) Molecules associated with innate immunity at different levels by immune subtype. Significant differences are illustrated as. (C) Estimation of 14 stromal cell types in six immune subtypes within the TME. (D-F) Expression, proportion of somatic copy number variants (SCNVs), and correlation between their expression and LF of the two largest differentially expressed genes across immune subtypes. (G) The expression of TNF and LTA is inversely correlated with methylation levels in breast cancer. (H) Expression regulation by miRNAs.

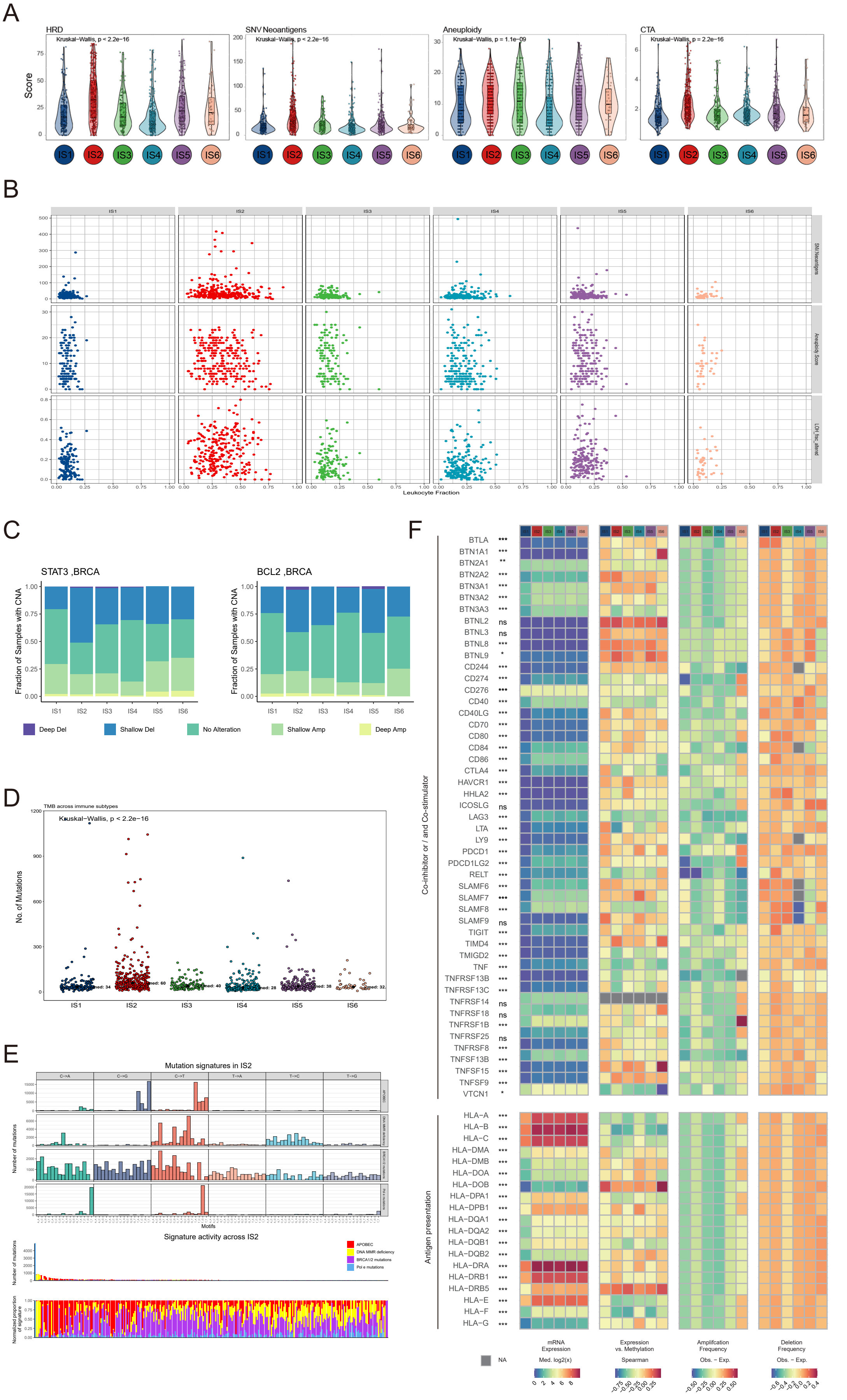

Fig S4. Genomic alterations and regulation of immunomodulators. (A) Scores of HRD, SNV neoantigens, aneuploidy and CTA by immune subtype. (B) Relationships between leukocyte fractions with neoantigen burden, aneuploidy, and number of segments with loss of heterozygosity (LOH) according to the immune subtype. (C) Proportion of SCNVs within STAT3 and BCL2. (D) Mutation burden according to the immune subtype. (E) Four mutation patterns identified by Bayesian NMF (upper panel); bar plot showing the total number of mutations with four mutation signatures (middle panel) and relative number of mutation types (lower panels). (F) From left to right: mRNA expression (median normalized expression), expression versus methylation (gene expression correlation with the DNA methylation beta-value), amplification frequency, and deletion frequency for regulators.

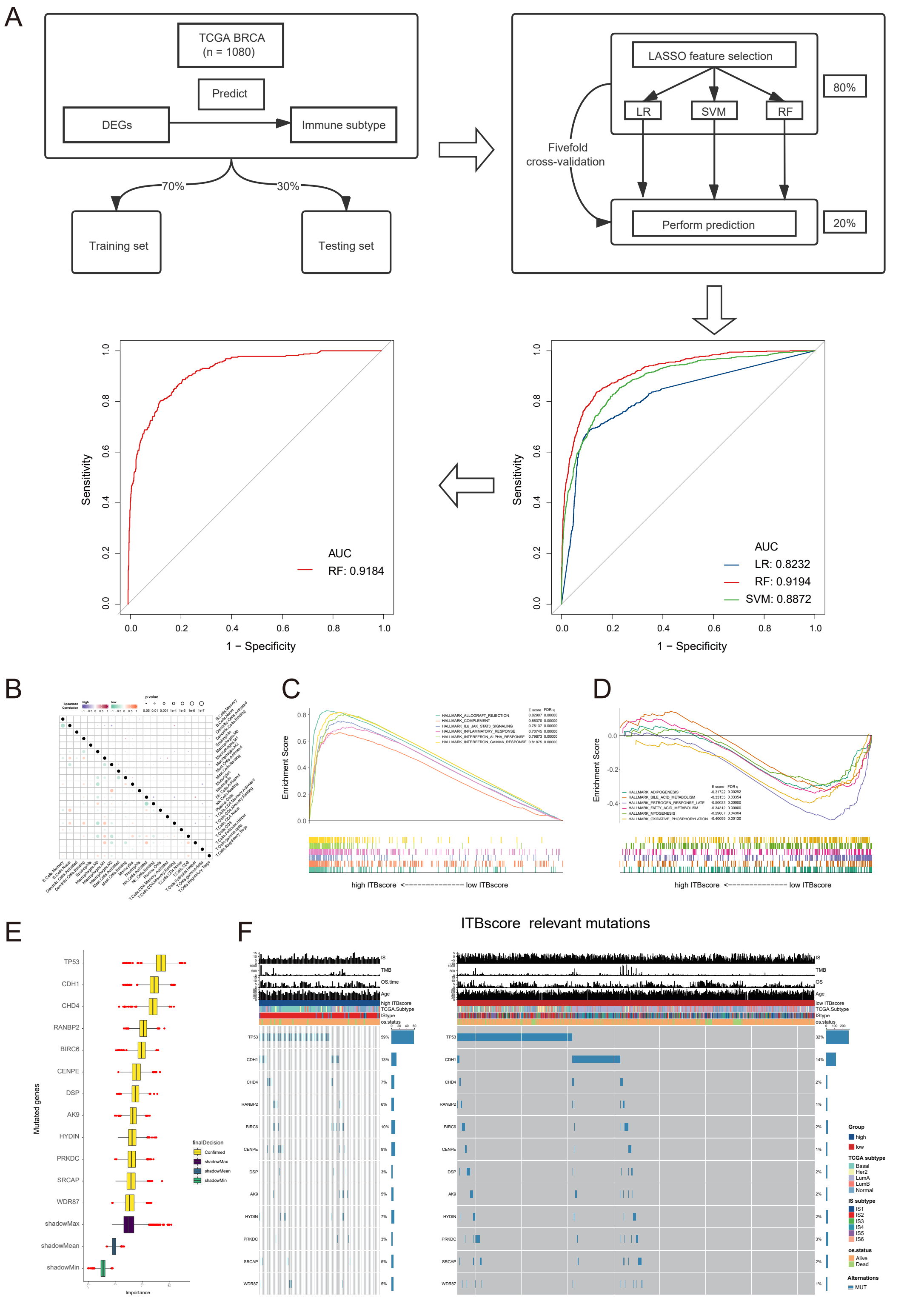

Fig S5. Characteristics of the immune prognostic signature. (A) Three machine learning methods were used to assess the power of DEGs to classify distinct immune subtypes. (B) Spearman correlation coefficients of 22 estimated immune cell types by CIBERSORT in the high (top) and low (bottom) ITBscore groups. (C-D) GSEA using the hallmark gene set in the high ITBscore group versus the low ITBscore group. (E) Identification of the top mutated genes using the random forest algorithm. (F) OncoPrint plot of patients with high ITBscores on the left (blue) and those with low ITBscores on the right (red).

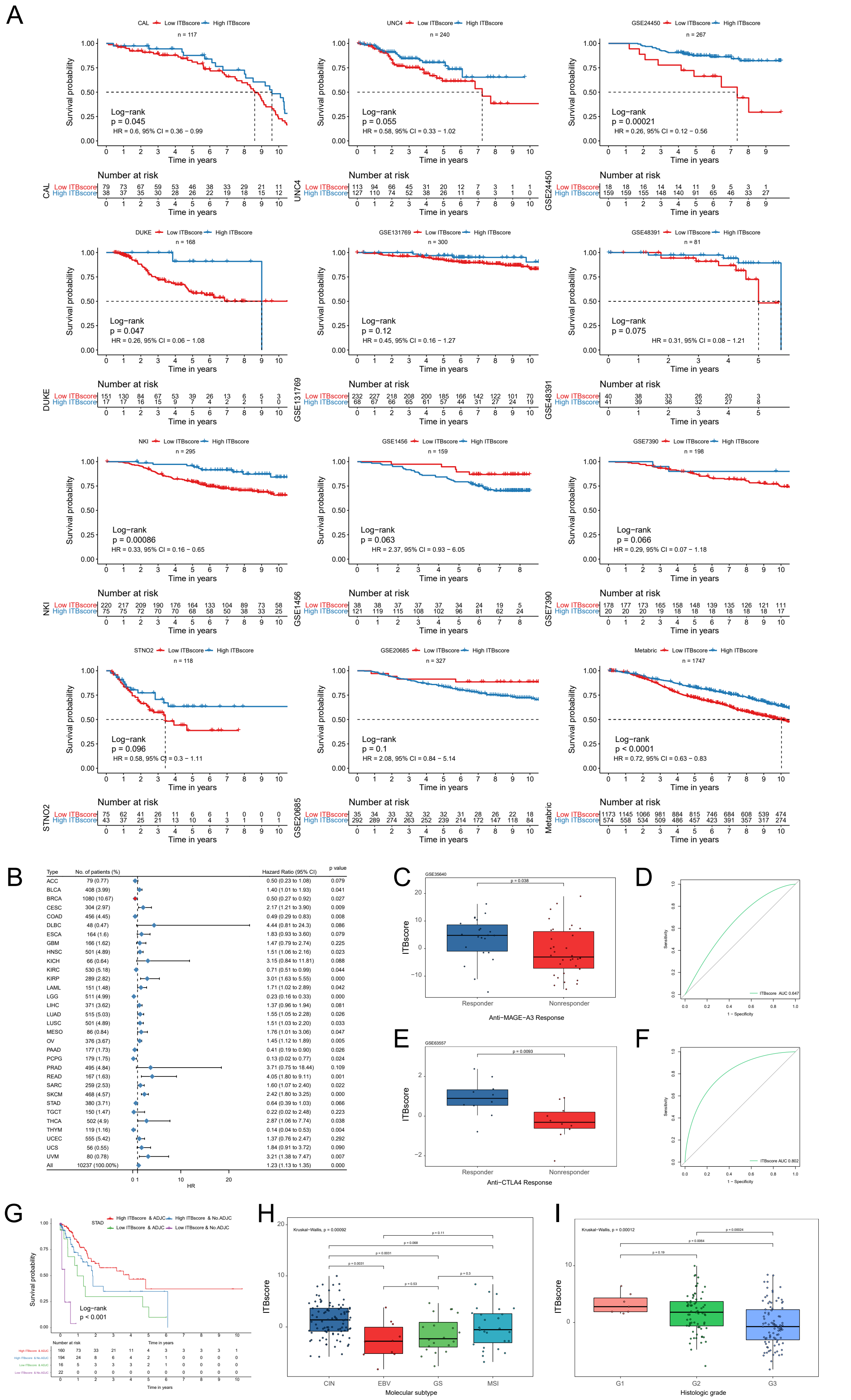

Fig S6. Validation of the clinical value of the ITBscore. (A) Prognostic value of the ITBscore in 12 independent breast cancer cohorts. (B) Pan-cancer analyses of the predictive value of the ITBscore. (C) Distribution of the ITBscore according to the anti–MAGE-A3 clinical response. (D) AUC value showing the predictive performance of the ITBscore in the GSE35640 cohort. (E) Distribution of the ITBscore according to the anti–CTLA−4 clinical response. (F) AUC value showing the predictive performance of the ITBscore in the GSE63557 cohort. (G) Kaplan–Meier curves for patients in the TCGA-STAD cohort stratified by both the receipt of adjuvant chemotherapy (ADJC) and ITBscore. (H) Distribution of the ITBscore among TCGA-STAD molecular subtypes. (I) Distribution of the ITBscore according to TNM stage in the TCGA-STAD cohort.
