# Supplementary material for: Immunogenomic Landscape in Breast Cancer Reveals Immunotherapeutically Relevant Gene Signatures
Source: Front Immunol. 2022 Jan 27;13:805184. doi: 10.3389/fimmu.2022.805184 (PMC8829007; doi:10.3389/fimmu.2022.805184)
Supplement: Supplementary file 3 [file DataSheet_3.docx]

Supplementary methods

Data collection and preprocessing

For the TCGA dataset, we acquired the standardized, batch and platform corrected data matrices and mutation data from the PanCancer Atlas consortium (https://gdc.cancer.gov/about-data/publications/pancanatlas), including gene expression, miRNA expression, gene mutations, copy number mutation and DNA methylation of 1080 breast cancer patients. The clinical data associated with TCGA patients were obtained from Liu et al. [1]. The molecular subtypes used in this study were available at http://bioinformaticsfmrp.github.io/TCGAbiolinks/subtypes.html. Scores involved in DNA damage repair deficiency were derived from Knijnenburg et al. [2], such as copy number burden, aneuploidy, loss of heterozygosity, and homologous recombination deficiency (HRD). The leukocyte fraction values, stromal fraction and neoantigens-related data were acquired from published research [3]. The gene expression data obtained as Fragments per Kilobase Million (FPKM) were transformed into Transcripts Per Million (TPM) to improve the accuracy. Raw counts for differential expression analysis and mutation data were downloaded and preprocessed using the TCGAbilinks R package [4]. Level 4 copy number calls from GISTIC2 [5] were acquired from GDAF Firehose in January 2016. For the Metabric dataset, gene expression, mutations and clinical data of 1999 breast cancer patients were interrogated from cBio Cancer Genomics Portal (www.cbioportal.org) [6-8]. For GEO datasets, the raw data of microarray datasets generated by Affymetrix (including GSE1456, GSE20685, GSE48391, GSE7390, GSE35640, and GSE63557) and Illumina (including GSE131769, GSE24450, and GSE78220), and corresponding clinical data were downloaded from GEO (https://www.ncbi.nlm.nih.gov/geo/). Robust Multi-array Average (RMA) algorithm was used to process the datasets from Affymetrix for background correction, normalization, and summarization in the Affy R package [9]. The raw datasets from Illumina were processed using the lumi software package. Furthermore, to investigate the datasets encompassing CAL, DUKE, NKI, STNO2, and UNC4, we downloaded the MetaGxData project from published research by Gendoo et al. [10]. MetaGxData project comprises curated transcriptomic data and standardized clinical, pathological, survival, and treatment metadata for breast cancer.

Preparation of functional and clinical gene signatures

We utilized 83 immune expression signatures, collected by Vésteinn et al. [3] from published studies related to the immune response in cancer [11-18], to characterize intratumoral immune states. Meantime, ten breast cancer signatures were compiled from published research in breast cancer [97-99], which involved proliferation, invasion/metastasis, apoptosis, ꞵ-catenin, prolactin signaling, E2F3 signaling, ER signaling, HER2 signaling, PTEN signaling and SRC signaling. Then, ten oncogenic pathways curated by the TCGA PanCancer Atlas Pathway subgroup [19] are also utilized in this study, including RTK/RAS pathway, NRF2 pathway, TGF beta pathway, PI3K pathway, WNT pathway, MYC pathway, TP53 pathway, cell cycle pathway, HIPPO pathway and NOTCH pathway. Furthermore, fourteen signatures related to DNA repair pathways were collected from the Wood lab [20-22]. Finally, we curated clinical signatures for estimating the clinical prognosis [23-27]. The limma R package was used to defined the difference [28]. The BH-adjusted p-value < 0.05 was regarded as the differentially expressed genes in each cancer type.

Genomic alterations in signaling pathways and functional signatures

To discover the potential pathways between subtypes of BRCA, the gene set variation analysis (GSVA) [29] was performed with Kyoto Encyclopedia of Genes and Genomes (KEGG) [30] gene sets from the Molecular Signature Database (MSigDB, c2.cp.kegg.v7.1.symbols.gmt), as implemented in the GSVA R package. A specific subtype's variations against the rest samples were estimated using the DIDS, an R package to identify imbalanced differential genes in heterogeneous populations [31]. The results were assumed significant if their Wilcoxon BH p-value were below 0.05. Furthermore, the Kruskal-Wallis test was used to compare six immune subtypes. BH-adjusted p < 0.05 was significant.

We further investigated the aberrations of immune signatures, breast cancer signatures, oncogenic signaling pathways and DNA repair signatures using ssGSEA over immune subtypes. The connection of the genomically-altered pathways and signatures in each immune subgroup was valued. The relationship was presumed significant if their BH-adjusted p-value were below 0.05.

Estimation of cell abundance in the tumor microenvironment

To explore the association of immune subtypes and cell fractions in the tumor microenvironment (TME), we adopted three strategies: first, CIBERSORT [32] compiled by deconvolution algorithm and LM22 signature were used quantify the proportions of immune cells. LM22 signature is composed of 547 genes and enables exact and specific distinction of 22 human hematopoietic cell phenotypes; second, due to the gap of CIBERSORT in stromal cells quantification, we performed the ssGSEA method to calculate the relative abundance of stromal cells in each sample with gene expression data. Gene sets were referred from published research involved in 14 signatures of stromal cells [33]; third, by collecting gene set from Angelova et al. [34], some key cell types in TME were also evaluated using ssGSEA as mentioned above, such as T helper 1, 2 and 17 cells (Th1, Th2, and Th17) and myeloid-derived suppressor cells (MDSC). To explore the distribution of major classes of immune cells (from CIBERSORT) across subtypes, we defined the the aggregation as follows: Lymphocytes = B cells naive + B cells memory + T cells CD4 naive + T cells CD4 memory resting + T cells CD4 memory activated + T cells follicular helper + T cells regulatory + Tregs + T cells gamma delta + T cells CD8 + NK cells resting + NK cells activated + Plasma cells; Macrophages = Monocytes + Macrophages M0 + Macrophages M1 + Macrophages M2; Dendritic cells = Dendritic cells resting + Dendritic cells activated; Mast cells = Mast cells resting + Mast cells activated; Neutrophils = Neutrophils; Eosinophils = Eosinophils [3].

Correlation between gene expression and DNA methylation

To compare the differential mRNA expression across immune subtypes, the samples with missing expression values were removed. Normalized mRNA expression (log_2_(x + 1) were used to check for batch effects. Kruskal-Wallis tests were executed to inspect the differences with adjusting p values by the BH method.

To study the correlation between gene expression and DNA methylation, DNA methylation probes from Illumina's 450k and 27k were mapped to target genes using IlluminaHumanMethylation450kanno.ilmn12.hg19 and IlluminaHuman Methylation27kanno.ilmn12.hg19 R packages. Spearman correlations were computed within each immune subtype. The mean correlation value was made if multiple probes were associated with one gene.

Prediction of miRNA for immunomodulators

Spearman correlation of microRNA (miRNA) and corresponding gene expression was evaluated using normalized and batch corrected expression matrix of 743 miRNA genes. Analysis for miRNA target prediction was performed in the miRDB database (http://www.mirdb.org/) [35] with the HGNC gene symbol as input.

Exploration for subtype-specific mutations

We enrolled genes with mutation frequency greater than 2.0% to analysis immune subtype-specific mutated genes. To impair confounding effects, we performed logistic regression model analysis based on mutation status of target genes (ignoring silent events) and tumor mutation load fitting [36]. Permutation test adopted from glmperm R package [37] was used to assess the significant difference within one subtype compared with others. P value below 0.05 after adjusting for mutation load was represented significant.

Copy number variation

Prior to evaluating copy number variation, we defined variation types using deep amplifications, shallow amplifications, non-alterations, shallow deletions, and deep deletions according to the CN value of GISTIC2.0 (+2, +1, 0, -1 and -2) [3].

We utilized the data matrices managed by PanCan GISTIC2.0 on the ISAR-corrected Affymetrix genome-wide human SNP6.0 array to evaluate the copy number values. The proportions of various types in each sample were estimated across immune subtypes. The difference between observing and expected frequencies was also calculated by the median value for each gene.

Differentially expressed analysis across the immune subtypes

We aggregated patients based on the representative immune signatures to identify associated genes of a given immune subtype compared with others. Differentially expressed genes (DEGs) were analyzed using raw counts and the DESeq2 R package [38] with statistical methods modeled with the negative binomial distribution. The p values were adjusted according to the BH approach for controlling the false discovery rate (FDR). Genes with FDR < 0.05 were assigned as significant.

Reference

1. Liu J, Lichtenberg T, Hoadley KA, Poisson LM, Lazar AJ, Cherniack AD, Kovatich AJ, Benz CC, Levine DA, Lee AV, et al: **An Integrated TCGA Pan-Cancer Clinical Data Resource to Drive High-Quality Survival Outcome Analytics.** *Cell* 2018, **173:**400-416 e411.

2. Knijnenburg TA, Wang L, Zimmermann MT, Chambwe N, Gao GF, Cherniack AD, Fan H, Shen H, Way GP, Greene CS, et al: **Genomic and Molecular Landscape of DNA Damage Repair Deficiency across The Cancer Genome Atlas.** *Cell Rep* 2018, **23:**239-254 e236.

3. Thorsson V, Gibbs DL, Brown SD, Wolf D, Bortone DS, Ou Yang TH, Porta-Pardo E, Gao GF, Plaisier CL, Eddy JA, et al: **The Immune Landscape of Cancer.** *Immunity* 2018, **48:**812-830 e814.

4. Colaprico A, Silva TC, Olsen C, Garofano L, Cava C, Garolini D, Sabedot TS, Malta TM, Pagnotta SM, Castiglioni I, et al: **TCGAbiolinks: an R/Bioconductor package for integrative analysis of TCGA data.** *Nucleic Acids Res* 2016, **44:**e71.

5. Mermel CH, Schumacher SE, Hill B, Meyerson ML, Beroukhim R, Getz G: **GISTIC2.0 facilitates sensitive and confident localization of the targets of focal somatic copy-number alteration in human cancers.** *Genome Biol* 2011, **12:**R41.

6. Curtis C, Shah SP, Chin SF, Turashvili G, Rueda OM, Dunning MJ, Speed D, Lynch AG, Samarajiwa S, Yuan Y, et al: **The genomic and transcriptomic architecture of 2,000 breast tumours reveals novel subgroups.** *Nature* 2012, **486:**346-352.

7. Cerami E, Gao J, Dogrusoz U, Gross BE, Sumer SO, Aksoy BA, Jacobsen A, Byrne CJ, Heuer ML, Larsson E, et al: **The cBio cancer genomics portal: an open platform for exploring multidimensional cancer genomics data.** *Cancer Discov* 2012, **2:**401-404.

8. Gao J, Aksoy BA, Dogrusoz U, Dresdner G, Gross B, Sumer SO, Sun Y, Jacobsen A, Sinha R, Larsson E, et al: **Integrative analysis of complex cancer genomics and clinical profiles using the cBioPortal.** *Sci Signal* 2013, **6:**pl1.

9. Gautier L, Cope L, Bolstad BM, Irizarry RA: **affy--analysis of Affymetrix GeneChip data at the probe level.** *Bioinformatics* 2004, **20:**307-315.

10. Gendoo DMA, Zon M, Sandhu V, Manem VSK, Ratanasirigulchai N, Chen GM, Waldron L, Haibe-Kains B: **MetaGxData: Clinically Annotated Breast, Ovarian and Pancreatic Cancer Datasets and their Use in Generating a Multi-Cancer Gene Signature.** *Sci Rep* 2019, **9:**8770.

11. Cheng WY, Ou Yang TH, Anastassiou D: **Biomolecular events in cancer revealed by attractor metagenes.** *PLoS Comput Biol* 2013, **9:**e1002920.

12. Cheng WY, Ou Yang TH, Anastassiou D: **Development of a prognostic model for breast cancer survival in an open challenge environment.** *Sci Transl Med* 2013, **5:**181ra150.

13. Wolf DM, Lenburg ME, Yau C, Boudreau A, van 't Veer LJ: **Gene co-expression modules as clinically relevant hallmarks of breast cancer diversity.** *PLoS One* 2014, **9:**e88309.

14. Galon J, Angell HK, Bedognetti D, Marincola FM: **The continuum of cancer immunosurveillance: prognostic, predictive, and mechanistic signatures.** *Immunity* 2013, **39:**11-26.

15. Bedognetti D, Hendrickx W, Ceccarelli M, Miller LD, Seliger B: **Disentangling the relationship between tumor genetic programs and immune responsiveness.** *Curr Opin Immunol* 2016, **39:**150-158.

16. Gentles AJ, Newman AM, Liu CL, Bratman SV, Feng W, Kim D, Nair VS, Xu Y, Khuong A, Hoang CD, et al: **The prognostic landscape of genes and infiltrating immune cells across human cancers.** *Nat Med* 2015, **21:**938-945.

17. Godec J, Tan Y, Liberzon A, Tamayo P, Bhattacharya S, Butte AJ, Mesirov JP, Haining WN: **Compendium of Immune Signatures Identifies Conserved and Species-Specific Biology in Response to Inflammation.** *Immunity* 2016, **44:**194-206.

18. Subramanian A, Tamayo P, Mootha VK, Mukherjeed S, Ebert BL, Gillette MA, Paulovich A, Pomeroy SL, Golub TR, Lander ES, Mesirov JP: **Gene set enrichment analysis: A knowledge-based approach for interpreting genome-wide expression profiles.** *PNAS* 2005, **102:**15545-15550.

19. Sanchez-Vega F, Mina M, Armenia J, Chatila WK, Luna A, La KC, Dimitriadoy S, Liu DL, Kantheti HS, Saghafinia S, et al: **Oncogenic Signaling Pathways in The Cancer Genome Atlas.** *Cell* 2018, **173:**321-337.e310.

20. ddddAravind L, Walker DR, Koonin EV: **Conserved domains in DNA repair proteins and evolution of repair systems.** *Nucleic Acids Res* 1999, **27:**1223-1242.

21. Wood RD, Mitchell M, Sgouros J, Lindahl T: **Human DNA repair genes.** *Science* 2001, **291:**1284-1289.

22. Lange SS, Takata K, Wood RD: **DNA polymerases and cancer.** *Nat Rev Cancer* 2011, **11:**96-110.

23. Criscitiello C, Bayar MA, Curigliano G, Symmans FW, Desmedt C, Bonnefoi H, Sinn B, Pruneri G, Vicier C, Pierga JY, et al: **A gene signature to predict high tumor-infiltrating lymphocytes after neoadjuvant chemotherapy and outcome in patients with triple-negative breast cancer.** *Ann Oncol* 2018, **29:**162-169.

24. Akcakanat A, Zhang L, Tsavachidis S, Meric-Bernstam F: **The rapamycin-regulated gene expression signature determines prognosis for breast cancer.** *Mol Cancer* 2009, **8:**75.

25. Tobin NP, Harrell JC, Lovrot J, Egyhazi Brage S, Frostvik Stolt M, Carlsson L, Einbeigi Z, Linderholm B, Loman N, Malmberg M, et al: **Molecular subtype and tumor characteristics of breast cancer metastases as assessed by gene expression significantly influence patient post-relapse survival.** *Ann Oncol* 2015, **26:**81-88.

26. Sota Y, Naoi Y, Tsunashima R, Kagara N, Shimazu K, Maruyama N, Shimomura A, Shimoda M, Kishi K, Baba Y, et al: **Construction of novel immune-related signature for prediction of pathological complete response to neoadjuvant chemotherapy in human breast cancer.** *Ann Oncol* 2014, **25:**100-106.

27. Werner S, Brors B, Eick J, Marques E, Pogenberg V, Parret A, Kemming D, Wood AW, Edgren H, Neubauer H, et al: **Suppression of early hematogenous dissemination of human breast cancer cells to bone marrow by retinoic Acid-induced 2.** *Cancer Discov* 2015, **5:**506-519.

28. Ritchie ME, Phipson B, Wu D, Hu Y, Law CW, Shi W, Smyth GK: **limma powers differential expression analyses for RNA-sequencing and microarray studies.** *Nucleic Acids Res* 2015, **43:**e47.

29. Hänzelmann S, Castelo R, Guinney J: **GSVA: gene set variation analysis for microarray and RNA-seq data.** *BMC Bioinformatics* 2013, **14:**7.

30. Kanehisa M, Sato Y, Kawashima M, Furumichi M, Tanabe M: **KEGG as a reference resource for gene and protein annotation.** *Nucleic Acids Res* 2016, **44:**D457-462.

31. de Ronde JJ, Rigaill G, Rottenberg S, Rodenhuis S, Wessels LF: **Identifying subgroup markers in heterogeneous populations.** *Nucleic Acids Res* 2013, **41:**e200.

32. Newman AM, Liu CL, Green MR, Gentles AJ, Feng W, Xu Y, Hoang CD, Diehn M, Alizadeh AA: **Robust enumeration of cell subsets from tissue expression profiles.** *Nat Methods* 2015, **12:**453-457.

33. Aran D, Hu Z, Butte AJ: **xCell: digitally portraying the tissue cellular heterogeneity landscape.** *Genome Biology* 2017, **18**.

34. Angelova M, Charoentong P, Hackl H, Fischer ML, Snajder R, Krogsdam AM, Waldner MJ, Bindea G, Mlecnik B, Galon J, Trajanoski Z: **Characterization of the immunophenotypes and antigenomes of colorectal cancers reveals distinct tumor escape mechanisms and novel targets for immunotherapy.** *Genome Biol* 2015, **16:**64.

35. Chen Y, Wang X: **miRDB: an online database for prediction of functional microRNA targets.** *Nucleic Acids Res* 2020, **48:**D127-d131.

36. Xiao Y, Ma D, Zhao S, Suo C, Shi J, Xue MZ, Ruan M, Wang H, Zhao J, Li Q, et al: **Multi-Omics Profiling Reveals Distinct Microenvironment Characterization and Suggests Immune Escape Mechanisms of Triple-Negative Breast Cancer.** *Clin Cancer Res* 2019, **25:**5002-5014.

37. Werft W, Benner A: **glmperm: A Permutation of Regressor Residuals Test for Inference in Generalized Linear Models.** *R Journal* 2010, **2:**39-43.

38. Anders S, Huber W: **Differential expression analysis for sequence count data.** *Genome Biol* 2010, **11:**R106.
